# HuoXue QianYang QuTan Recipe attenuates left ventricular hypertrophy in obese hypertensive rats by improving mitochondrial function through SIRT1/PGC-1α deacetylation pathway

**DOI:** 10.1042/BSR20192909

**Published:** 2019-12-17

**Authors:** Jing Wang, Zhen-Hua Dong, Ming-Tai Gui, Lei Yao, Jian-Hua Li, Xun-Jie Zhou, De-Yu Fu

**Affiliations:** 1 Department of Cardiology, Yueyang Hospital of Integrated Traditional Chinese and Western Medicine Affiliated to Shanghai University of Traditional Chinese Medicine, Shanghai 200437, China; 2 Jinyang Community Health Center, Shanghai 200136, China

**Keywords:** HuoXue QianYang QuTan Recipe, Left ventricular hypertrophy, Mitochondrial function, Obesity hypertension, SIRT1/PGC-1α deacetylation

## Abstract

Mitochondrial dysfunction plays a vital role in the progression of left ventricular hypertrophy (LVH). Previous studies have confirmed that the disorder of SIRT1/PGC-1α deacetylation pathway aggravated mitochondrial dysfunction. HuoXue QianYang QuTan Recipe (HQQR) is a commonly used prescription that has shown therapeutic effects on obesity hypertension and its complications. However, the potential mechanisms are still unclear. In the present study, obesity hypertension (OBH) was established in rats and we investigated the efficacy and mechanisms of HQQR on LVH. Rats were divided into the five groups: (1) WKY-ND group, (2) SHR-ND group, (3) OBH-HF group, (4) OBH-HF/V group and (5) OBH-HF/H group. We evaluated body weight, Lee index and blood pressure (BP) before and every 2 weeks after treatment. After 10 weeks of treatment, we mainly detected glycolipid metabolic index, the severity of LVH, mitochondrial function along with SIRT1/PGC-1α deacetylation pathway. Our results showed that HQQR significantly lowered body weight, Lee index, BP and improved the disorder of glycolipid metabolism in OBH rats. Importantly, we uncovered HQQR could alleviate mitochondrial dysfunction in OBH rats by regulating SIRT1/PGC-1α deacetylation pathway. These changes could be associated with the inhibition of LVH.

## Introduction

The epidemic of obesity parallels that of hypertension [1]. Obesity exacerbates the course of hypertension and cardiovascular complications, which imposes a heavy burden on individuals, societies and healthcare systems worldwide [2,3]. Left ventricular hypertrophy (LVH), a strong risk factor for development from hypertension to adverse cardiovascular outcomes [4], determines overall risk stratification of hypertension, which is an effective therapeutic goal in controlling hypertension [5]. Furthermore, recent evidence also showed that obesity was a hemodynamically volume overload disease that resulted in alterations in cardiac morphology and ventricular function [1,6]. Obesity and hypertension produce interactive and additive effects on LVH [5]. With the global obesity and obesity-related diseases epidemic, LVH of obesity hypertension remains to be solved urgently.

Mitochondria are recognized as the main source of reactive oxygen species (ROS) as well as the primary targets of ROS attack associated with cardiac hypertrophy [7]. Myocardial mitochondria are susceptible to oxidative stress, which leads to impairment of mitochondrial function and pathological cardiac hypertrophy [8]. Additionally, disorder of mitochondrial biogenesis is an important pathological phenomenon of cardiac hypertrophy [9]. Therefore, maintaining mitochondrial function and integrity has become a potential therapeutic method to treat pathological cardiac hypertrophy [10].

Sirtuin 1 (SIRT1), a conserved NAD^+^-dependent protein deacetylase, is the upstream regulator of peroxisome proliferator-activated receptor γ co-activator 1α (PGC-1α) [11]. SIRT1 could protect cardiomyocytes from oxidative stress by deacetylating and activating PGC-1α [12,13]. Previous studies have confirmed that the disorder of SIRT1/PGC-1α deacetylation pathway increased oxidative stress and mitochondrial dysfunction [14]. Importantly, activation of SIRT1/PGC-1α deacetylation improved mitochondrial biogenesis by regulating nuclear respiratory factor-1 (NRF1) and mitochondrial transcription factor A (TFAM), which was beneficial to the maintenance of mitochondrial function [15,16]. Cui et al. reported that erythropoietin (EPO) improved mitochondrial function through SIRT1/PGC-1α deacetylation-mediated mitochondrial biogenesis and protected against doxorubicin (DOX)-induced cardiotoxicity [15]. Fang et al*.* verified that resveratrol (RSV) alleviated diabetic cardiac damage by alleviating oxidative stress and mitochondrial dysfunction associated with the regulation of SIRT1 on PGC-1α deacetylation [17]. Therefore, up-regulation of SIRT1/PGC-1α deacetylation pathway could be an attractive target for intervention and treatment of pathological cardiac hypertrophy [18–20].

In China, it has been well known that Chinese herbal medicine is effective in treating LVH [21]. Clinically, HuoXue QianYang QuTan Recipe (HQQR), a compound traditional Chinese herbal medicine, could lower blood pressure (BP), improve lipid metabolism and insulin resistance and reverse LVH among hypertensive patients with obesity. However, its specific molecular mechanisms under the efficacy have not been fully elucidated. Based on the above evidences, the present study was designed to explore whether HQQR could alleviate LVH in obese hypertensive rats by improving mitochondrial function through SIRT1/PGC-1α deacetylation pathway.

## Materials and methods

### Experimental animals and drugs

About 120 five-week-old male spontaneously hypertensive rats (SHR) (body weight, 150 ± 20 g) and 12 age/sex-matched Wistar-Kyoto rats (WKY) (body weight, 150 ± 20 g) were obtained from the Vital River Laboratory Animal Technology Co., Ltd (Animal license number: SCXK (Beijing) 2016-0006). The animal work was performed in the experimental animal center of Yueyang Hospital of Integrated Traditional Chinese and Western Medicine under a 12/12-h light/dark period cycle at controlled temperature (22 ± 2°C). The animal procedures were approved by the Institutional Animal Care and Use Committee at Yueyang Hospital of Integrated Traditional Chinese and Western Medicine Affiliated to Shanghai University of Traditional Chinese Medicine in accordance with the principles outlined in the NIH Guidelines for the Care and Use of Laboratory Animals.

Valsartan Capsule (batch number: H20040217) was manufactured by Novartis Pharmaceutical Factory (Beijing, China).

HQQR consists of *Salvia miltiorrhiza* 15 g, *Stone Cassia* 30 g, *Ligusticum chuanxiong* 9 g, *Uncaria angustifolia* 15 g, *Mulberry parasite* 15 g, *Hawthorn* 15 g and *Corn whisker* 30 g. We entrusted the Pharmacy Department of the General Hospital of Nanjing Military Region to purchase, mix, decoct, filter, concentrate and dry the seven herbs and get extraction powder according to the production process. Finally we got 3.45 kg powder, containing 5.98 g of crude medicine per gram.

### Experimental procedure

After acclimatized for 1 week, 108 SHR were randomly selected to feed high-fat diet (HFD), the other 12 and 12 WKY were fed normal diet. The normal diet (NO.P1101F) and HFD were purchased from Shanghai Puluteng Biotechnology Co., Ltd. The HFD consisted of 60% normal feed, 12% lard, 5% sucrose, 10% yolk powder, 3% milk powder, 4.7% raw peanut, 1% sesame oil, 2% salt, 2% cholesterol and 0.3% bile salt. After intervened for 10 weeks, rats with weight in the upper third of the SHR fed HFD were selected as obese hypertensive rats (OBH rats) [22]. Then rats were divided into five groups: WKY fed normal diet (WKY-ND, *n* = 12), SHR fed normal diet (SHR-ND, *n* = 12), OBH rats fed HFD (OBH-HF, *n* = 12), OBH rats fed HFD and given 30 mg/kg/d of valsartan by gavage (OBH-HF/V, *n* = 12), OBH rats fed HFD and given 38.7g/kg/d of HQQR crude drug by gavage (OBH-HF/H, *n* = 12). The drugs were given at 9:00 am every morning and administered for 10 weeks.

### Physiological and biochemical measurements

Body weight and body length of rats were measured before and every 2 weeks after treatment.

Lee index can be used to evaluate the degree of obesity in rats. Lee index was calculated as follows: Lee index = $\sqrt[{3}]{body\;weight\;(g)}$ × 10^3^/body length (cm) [23].

### BP including systolic blood pressure (SBP) and diastolic blood pressure (DBP)

BP including systolic blood pressure (SBP) and diastolic blood pressure (DBP) was measured before and every 2 weeks after treatment using tail-cuff method with intelligent non-invasive sphygmomanometer (BP-98A, Beijing Softron Biotechnology Co., Ltd, Beijing, China). Conscious and calm rats were fixed in 37°C preheat insulation barrels and the instrument parameters were set. Put the rat tail inside the tail cuff, started measuring, repeated the measurement 3–5 times, took the average and recorded. After treatment for 10 weeks, abdominal aortic blood was taken from the rats under anesthesia with sodium pentobarbital (40 mg/kg body weight, i.p.) and then centrifuged at 3000 rpm for 8 min. The supernatants were stored at −80°C. The left ventricular tissues were collected for morphological, biochemical and molecular examination and the rats were killed by cervical dislocation. Serum total cholesterol (TC), total triglycerides (TG), high-density lipoprotein-cholesterol (HDL-C), low-density lipoprotein-cholesterol (LDL-C) and fasting blood glucose (FBG) were assayed by automatic biochemical analyzer (Zhuoyue450, Kehua Bioengineering Co., Ltd, Shanghai, China). The level of fasting insulin (FIN) was detected by Rat FIN ELISA Kit (Shanghai Yuanye Biotechnology Co., Ltd, China). Homeostasis model assessment for the insulin resistance (HOMA-IR) index was calculated as follows: [FIN (mU/l) × FBG (mmol/l)]/22.5 [24].

### Assessment of left ventricle mass index (LVMI)

After abdominal aortic blood collected, their heart tissues were taken out rapidly, washed with precooled saline and dried by filter paper. Left ventricle tissues were separated and weighed. The LVMI was calculated as follows: left ventricle mass (mg)/body weight (g).

### Histopathological examination

Left ventricle tissues were taken and fixed with 4% paraformaldehyde for 24 h and then dehydrated by an automatic dehydrator and embedded in paraffin the next day. Sections (5 μm thick) were operated for hematoxylin and eosin (H&E) staining according to the standard protocol. The stained sections were placed under an optical microscope (Leica, Germany), and five visual fields were selected for each sample to be photographed at 400× magnification for morphological observation and analysis. Additionally, in order to display the outline of cardiomyocytes intuitively, we did wheat germ agglutinin (WGA) staining. After embedding left ventricle tissues into paraffin, sections (5 μm thick) were used for WGA staining according to the standard protocol. Cardiomyocyte cross-sectional area was observed by fluorescence microscope (Leica, Germany), and five visual areas were randomly selected in each section and examined at 400× magnification. The area of cardiomyocyte cross-section was calculated by ImageJ.

### Ultrastructure of mitochondria

Five 1 cubic millimeter of heart tissues at the apex of the left ventricles were separated and fixed in 2.5% glutaraldehyde for 2 h at 4°C, then washed with phosphate buffer, post-fixed in 1% osmic acid for 2 h at 4°C, washed continuedly, gradient dehydration by ethanol, soaked in acetone and embedded in epoxies at 37°C for 12 h, 60°C for 48 h. After ultrathin sections were cut at 50 nm and uranyl acetate-lead citrate staining was performed, the ultrastructure was observed by transmission electron microscope (FEI Tecnai G2 Spirit, Thermo Fisher Scientific, U.S.A.) and four visual areas of each sample were observed at 8200× magnification.

### Measurement of myocardial ATP content

The ATP content was measured using a ATP determination Kit (Nanjing Jiancheng Bioengineering Institute, Nanjing, China). The left ventricle tissues were homogenized and boiled to ensure the full release of ATP. After centrifugation, the ATP content of the supernatant was detected according to the protocol of the manufacturer and calculated as micromoles per gram of protein.

### Real-time PCR analysis

Total RNA was extracted from left ventricle tissues using Trizol reagent (Beyotime, Shanghai, China) and reverse transcribed to cDNA using a TaqMan RNA Reverse Transcription Kit (Takara, Dalian, China) according to manufacturer’s protocol. Then, qPCR was performed on an ABI Step One Plus Real-Time PCR System (Applied Biosystems, Foster City, U.S.A.) with SYBR Green Master Mix (Yeasen, Shanghai, China) according to the instructions provided with the kit. Relative expression was determined by the 2^−ΔΔ*C*_T_^ method. GAPDH was used as an internal control. The reaction conditions were as follows: 95°C for 5 min, followed by 45 cycles of 20 s at 90°C, 20 s at 60°C and 20 s at 72°C. The primer sequences were presented as follows: 5′- CTCCTTCTCCATCACCAAG-3′ and 5′-AAGAAGGCAGATCTATCG-3′ for A-type natriuretic peptide (ANP); 5′-CCAACACCAACCTGTCCAA-3′ and 5′-ACTCTTCATTCAGGCCCTTG-3′ for β-myosin heavy chain (β-MHC); 5′- GGCACAGTCAAGGCTGAGAATG-3′ and 5′-ATGGTGGTGAAGACGCCAGTA-3′ for GAPDH. DNA extraction from left ventricular tissues was performed, and the mitochondrial DNA (mtDNA) level of COX1 was determined. The primer sequences were presented as follows: 5′-CACATGAGCAAAAGCCCACT-3′ and 5′-ACGGCCGTAAGTGAGATGAA-3′ for COX1; 5′-GAGAGGGAAATCGTGCGT-3′ and 5′-GGAGGAAGAGGATGCGG-3′ for β-actin.

### Evaluation of mitochondrial oxidative stress damage

Mitochondrial manganese superoxide dismutase (Mn-SOD) in mitochondrial fraction from left ventricle tissues was detected using a Tissue Mitochondria Isolation Kit (Beyotime, Shanghai, China) and a Mn-SOD Assay Kit (Beyotime, Shanghai, China) according to the provided protocols respectively. The activity of Mn-SOD was normalized to milligram protein.

### Co-immunoprecipitation analysis

Left ventricle tissues were lysed on ice for 15 min in lysis buffer. About 500 μg of total protein was incubated with anti-PGC-1α (518025, Santa Cruz, U.S.A.) on a rotating incubator overnight at 4°C. Afterwards, samples were followed by precipitation with 70 μl of protein A/G-Plus-Agarose (Santa Cruz, U.S.A.) for 4 h at 4°C. Normal IgG (Proteintech, U.S.A.) was used as an negative control. The precipitated complexes were washed in IP buffer and resuspended in 30 μl of 2× loading buffer and boiled for 5 min before Western blot assay was performed.

### Western blot analysis

Protein was extracted from left ventricle tissues using 0.2 ml precooled lysis buffer/20 mg tissue. Protein concentration was measured by BCA protein concentration assay kit (Beyotime, Shanghai, China), and the supernatant was denatured at 95°C for 5 min in Laemmli sample buffer. Samples were subjected to 8% or 10% SDS-PAGE gels. After electrophoresis, proteins were electro-transferred to a polyvinylidene difluoride membrane (Merck, U.S.A.), which was incubated in the blocking buffer (5% non-fat milk, 20 mM Tris-HCl, 150 mM NaCl, pH 8.0, 0.01% Tween 20) for 1 h at room temperature and was followed by incubation with anti-COX1 (ab203912, Abcam, U.K.), anti-ATPase6 (A8193, Abclonal, China), anti-SIRT1 (sc-74465, Santa Cruz, U.S.A.), anti-PGC-1α (sc-518025, Santa Cruz, U.S.A.), anti-NRF1 (ab175932, Abcam, U.K.) and anti-TFAM (sc-166965, Santa Cruz, U.S.A.) overnight at 4°C. Binding of the primary antibody was detected by an enhanced chemiluminescence method (BeyoECL Star, P0018A, Beyotime) using horseradish peroxidase-conjugated secondary antibodies (Beyotime, Shanghai, China). The quantification of protein expression was performed using ImageJ.

### Statistical analysis

All data were presented as mean ± standard deviation (SD). Data were analyzed by statistical software SPSS 25.0 (SPSS Ltd., U.S.A.). One-way ANOVA was used for general data analysis. The values of *P* < 0.05 and *P* < 0.01 were both considered to be statistically significant.

## Results

### Effects of HQQR on body weight, Lee index, BP and glucolipid metabolism index

**Figure 1:**
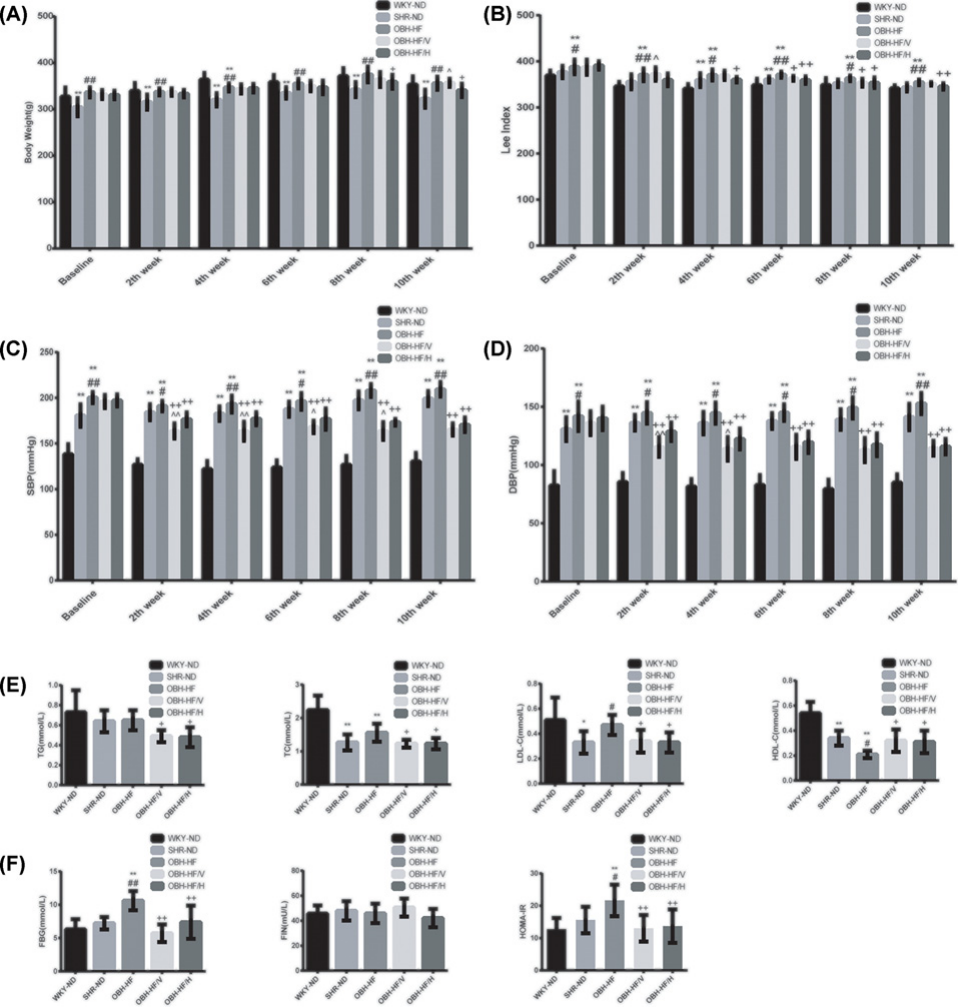
HQQR lowered body weight, Lee index, BP and improved glycolipid metabolism disorder in OBH rats (**A**–**D**) Body weight, Lee index and BP were measured before and every 2 weeks after treatment (*n* = 10–12 per group). (**E**) The level of lipid metabolism was assayed by automatic biochemical analyzer (*n* = 6 per group). (**F**) The level of glucose metabolism was assayed by automatic biochemical analyzer and ELISA (*n* = 6 per group). Data are mean ± SD. *P < 0.05, **P < 0.01 compared with WKY-ND; ^#^*P* < 0.05, ^##^*P* < 0.01 compared with SHR-ND; ^+^*P* < 0.05, ^++^*P* < 0.01 compared with OBH-HF; ^∧^*P* < 0.05, ^∧∧^*P* < 0.01 compared with OBH-HF/H.

In the present study, we found that after HFD treatment for 10 weeks, the body weight, Lee index and BP of OBH-HF were higher than those of WKY-ND and SHR-ND (*P* < 0.01 or *P* < 0.05). Compared with OBH-HF, HQQR could significantly reduce the body weight after treatment for 8 weeks and decreased the Lee index after treatment for 4 weeks, which both continued to decrease in the subsequent weeks (*P* < 0.01 or *P* < 0.05). These results indicated the beneficial effects of HQQR on alleviating obesity (Figure 1A,B). Starting from the second week, HQQR could significantly reduce both the SBP and DBP of OBH-HF/H until the end of treatment (*P* < 0.01), and the antihypertensive effect of HQQR was comparable to valsartan finally but more stable than that of valsartan (Figure 1C,D).

Obesity hypertension is also accompanied with glycolipid metabolism disorder. After HFD treatment for 20 weeks, the LDL-C level of OBH-HF was higher than that of SHR-ND (*P* < 0.05), while the HDL-C level of OBH-HF was lower than that of WKY-ND and SHR-ND (*P* < 0.01 or *P* < 0.05); moreover, compared with a normal diet, HFD could significantly increase the level of FBG and HOMA-IR index (*P* < 0.01 or *P* < 0.05). The above changes indicated that after HFD treatment for 20 weeks, there was obvious disorder of glycolipid metabolism and insulin resistance in OBH-HF. In addition, we found that after treatment for 10 weeks, HQQR could improve glycolipid metabolism evidenced by reducing the levels of TC, TG, LDL-C, FBG, HOMA-IR index (*P* < 0.01 or *P* < 0.05) and significantly increasing the level of HDL-C in OBH-HF/H (*P* < 0.05) (Figure 1E,F).

### HQQR alleviated LVH in OBH rats

**Figure 2:**
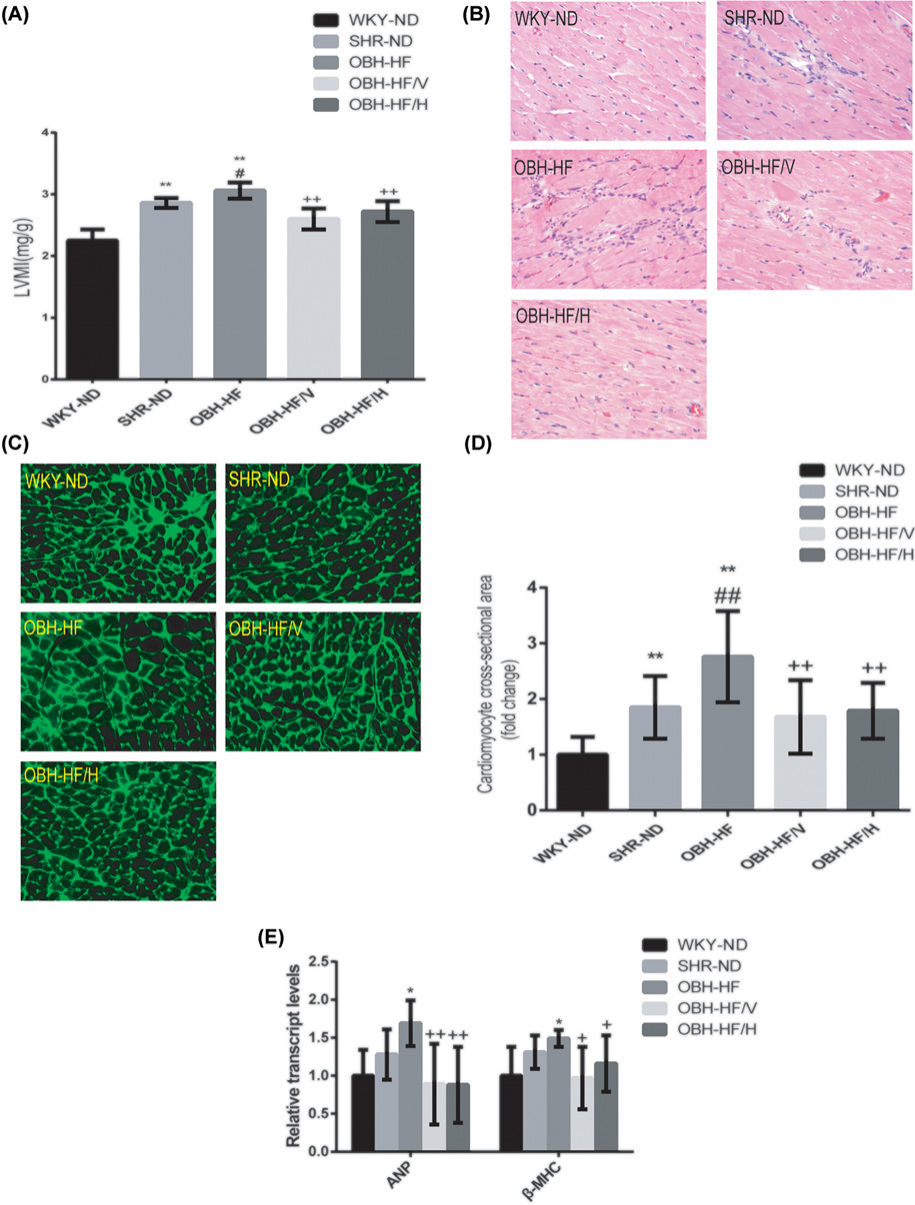
HQQR alleviated LVH in OBH rats (**A**) LVMI was calculated by left ventricle mass (mg)/body weight (g) (*n* = 6 per group). (**B**) Representative photomicrographs of HE staining; 400× magnification. (**C**) Representative photomicrographs of WGA staining; 400× magnification. (**D**) Quantification of cardiomyocyte cross-sectional area. (**E**) Real-time PCR analysis for the mRNA expression of ANP and β-MHC were performed. Relative fold change of ANP and β-MHC was presented in the graph (*n* = 6 per group). Data are mean ± SD. *P < 0.05, **P < 0.01 compared with WKY-ND; ^#^*P* < 0.05, ^##^*P* < 0.01 compared with SHR-ND; ^+^*P* < 0.05, ^++^*P* < 0.01 compared with OBH-HF.

Compared with WKY-ND and SHR-ND, OBH-HF had the highest LVMI and treatment with HQQR for 10 weeks could significantly decrease LVMI, which indicated that HFD could worsen LVH in SHR and HQQR could alleviated LVH (Figure 2A). By morphological examination, we found that OBH-HF showed a more severe pathological state of cardiac hypertrophy with swelling, enlargement and disordered cardiomyocytes, in which inflammatory cell infiltration could be seen. Interestingly, treatment with HQQR for 10 weeks markedly attenuated these pathological changes (Figure 2B).

Then we did WGA staining, showing that the cardiomyocyte cross-sectional area of OBH-HF was significantly larger than that of WKY-ND and SHR-ND (*P* < 0.01) and HQQR treatment for 10 weeks could significantly reduce the area (*P* < 0.01) (Figure 2C,D).

Finally, we also measured the mRNA expression of ANP and β-MHC, two hypertrophic biomarkers in left ventricle tissues, showing that compared with WKY- ND, the expression of ANP and β-MHC of OBH-HF were significantly up-regulated (*P* < 0.05) while could be down-regulated after HQQR treatment for 10 weeks (*P* < 0.01 or *P* < 0.05) (Figure 2E).

### HQQR improved the morphology of mitochondria and inhibited oxidative stress damage

**Figure 3:**
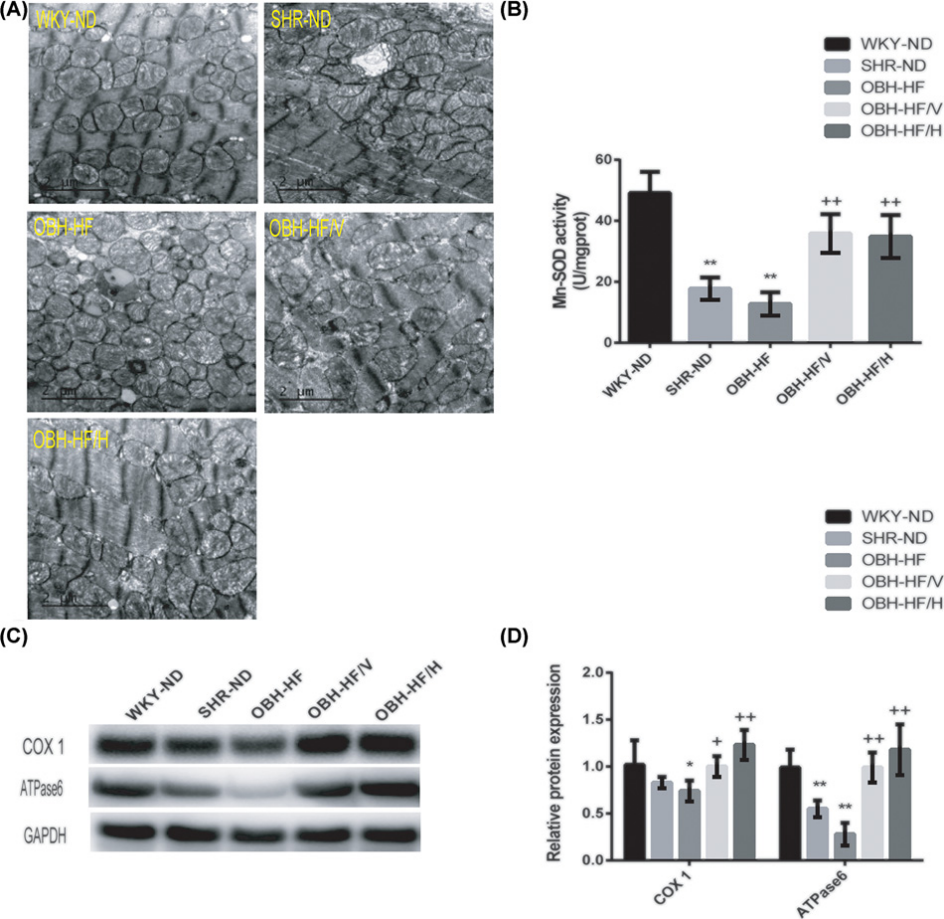
HQQR improved the morphology of mitochondria and inhibited oxidative stress damage (**A**) Representative photomicrographs of transmission electron microscope; 8200× magnification. (**B**) The level of the Mn-SOD activity was assayed in each group (*n* = 4 per group). (**C**) The protein expression of COX1 and ATPase6 was determined by immunoblotting. (**D**) Western blot quantification of COX1 and ATPase6 levels (*n* = 4 per group). Data are mean ± SD. *P < 0.05, **P < 0.01 compared with WKY-ND; ^+^*P* < 0.05, ^++^*P* < 0.01 compared with OBH-HF.

Mitochondrial injury plays an important role in the transition from compensated LVH to heart failure [25], we examined ultrastructure of the mitochondria using transmission electron microscopy. Compared with WKY-ND, the number of mitochondria of SHR-ND and OBH-HF increased compensatively in some parts, the muscle fibers were thicker, the morphology was irregular and swollen to varying degrees and some of them disintegrated. Moreover, the latter was more obvious. However, HQQR treatment for 10 weeks could alleviate injury with a better mitochondrial phenotype relatively (Figure 3A).

Under oxidative stress, the activity of endogenous antioxidant enzymes decreased. As expected, the Mn-SOD activity of SHR-ND and OBH-HF was obviously lower than that of WKY-ND (*P* < 0.01). However, HQQR treatment for 10 weeks abolished this change (*P* < 0.01) (Figure 3B).

Oxidative stress can induce cytotoxicity and damage in mitochondria, and mitochondrial-derived ROS is related to the leak of electrons from the electron transport chain (ETC) [26]. In order to detect ETC integrity, we measured the expression of COX1 and ATPase6, two electron transport chain subunits of mitochondrial complexes IV and V respectively. Western blot assay showed that the expression of COX1 and ATPase6 proteins was both down-regulated of OBH-HF (*P* < 0.01 or *P* < 0.05) than those of WKY-ND, while HQQR treatment for 10 weeks could markedly increase the expression of COX1 and ATPase6 (*P* < 0.01) (Figure 3C,D).

### HQQR alleviated LVH and improved mitochondrial function by SIRT1/PGC-1α deacetylation pathway

**Figure 4:**
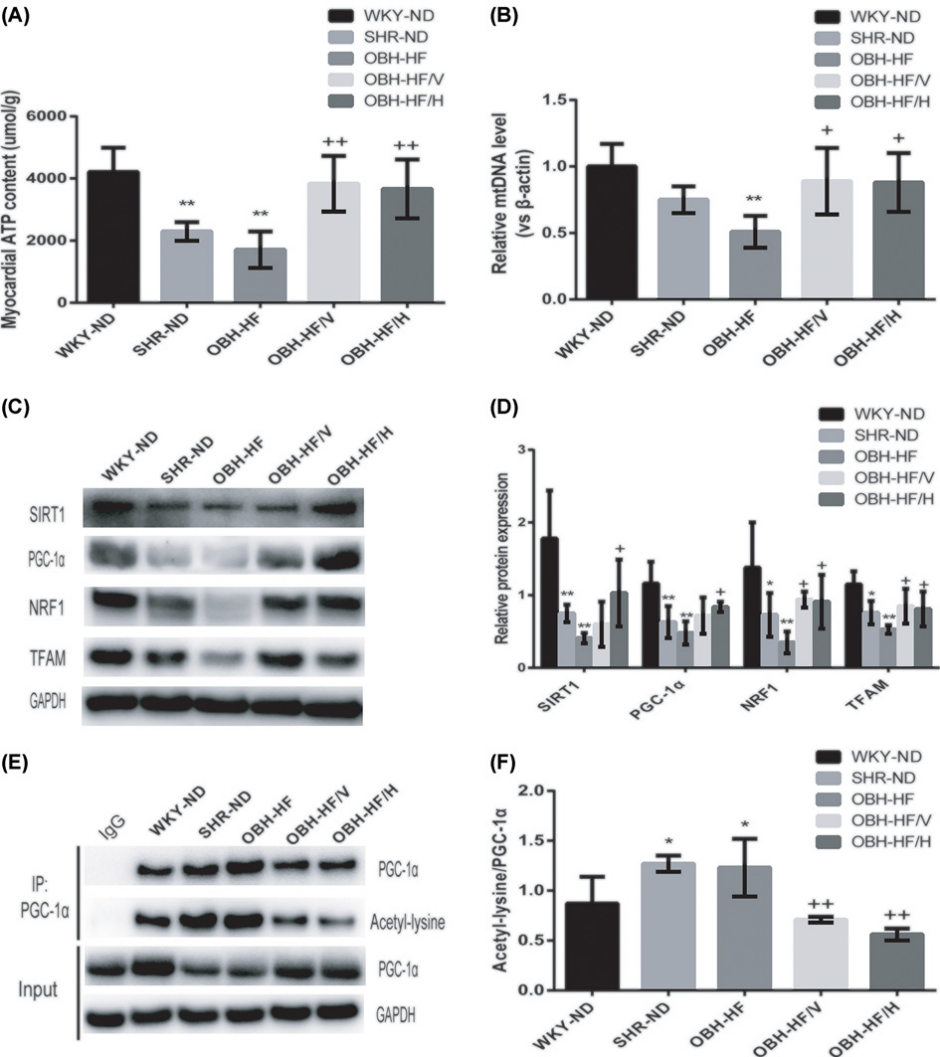
HQQR alleviated LVH and improved mitochondrial function by SIRT1/PGC-1α deacetylation pathway (**A**) The ATP content was detected in each group (*n* = 4 per group). (**B**) The mtDNA copy number was assayed in each group (*n* = 4 per group). (**C**) The protein expression of SIRT1, PGC-1α, NRF1 and TFAM was determined by immunoblotting. (**D**) Western blot quantification of SIRT1, PGC-1α, NRF1 and TFAM levels (*n* = 4 per group). (**E**) Immunoprecipitation and Western blot analysis of PGC-1α acetylation in each group. (**F**) The ratio of acetylated PGC-1α to precipitated PGC-1α protein was calculated (*n* = 4 per group). Data are mean ± SD. *P < 0.05, **P < 0.01 compared with WKY-ND; ^+^*P* < 0.05, ^++^*P* < 0.01 compared with OBH-HF.

During mitochondrial dysfunction, the level of ATP synthesis decreased, which was involved in the development of pathological cardiac hypertrophy [7]. We detected the myocardial ATP content to evaluate the mitochondrial function in regard to energy production. As expected, the ATP content of SHR-ND and OBH- HF was significantly lower than that of WKY-ND (*P* < 0.01), while HQQR administration for 10 weeks could markedly increase myocardial ATP content (*P* < 0.01) (Figure 4A).

Damage to mitochondrial biogenesis is one of the reasons for mitochondrial dysfunction [27], we examined the mtDNA copy number as assayed by the ratio of COX1 to β-actin (COX1/β-actin). The OBH-HF showed a reduction in the COX1/β-actin ratio compared with that of WKY-ND (*P* < 0.01). However, HQQR administration for 10 weeks could increase the mtDNA copy number (*P* < 0.05) (Figure 4B). Then, we measured four vital indicators regulating mitochondrial biogenesis. Western blot assay revealed that the expression of SIRT1, PGC-1α, NRF1 and TFAM proteins of OBH-HF was lower than those of WKY-ND (*P* < 0.01) while HQQR treatment for 10 weeks could up-regulate their protein levels (*P* < 0.05) (Figure 4C,D).

Previous studies have shown that SIRT1 was involved in the regulation of cellular responses via deacetylation of lysine residues such as FOXO1 and PGC-1α [28]. Deacetylation of PGC-1α by SIRT1 is considered to be an important regulatory mechanism of mitochondrial function and oxidative stress [29]. In the present study, to evaluate the expression level of acetylated PGC-1α protein, PGC-1α was immunoprecipitated and the acetyl group was determined by anti-acetyl antibody. We found that the acetylation of PGC-1α was markedly increased in OBH-HF than that of WKY-ND (*P* < 0.05), which also showed a upward trend compared with SHR-ND, and HQQR treatment for 10 weeks could eliminate the change (*P* < 0.01) (Figure 4E,F).

## Discussion

In the present study, we found that HQQR treatment for 10 weeks could markedly lower body weight, Lee index, BP and improve the disorder of glycolipid metabolism in OBH rats. Importantly, we uncovered HQQR could improve mitochondrial function in OBH rats by regulating SIRT1/PGC-1α deacetylation pathway. These changes could be associated with the inhibition of LVH.

Large-scale epidemiological studies have shown that obesity was closely related to the increase of blood pressure in patients with hypertension [30,31]. Weight gain of 10 kg increased SBP by 3 mmHg and DBP by 2.3 mmHg, which elevated the risk of coronary heart disease by 12% [32]. In addition, severe obesity has long been considered to be a powerful and independent predictor of left ventricular mass, left ventricular wall thickness, left ventricular diameter, left ventricular systolic function and diastolic function deterioration [2]. Furthermore, in chronic hypertension, elevated blood pressure led to thickening of the ventricular wall and functional changes to adapt to specific conditions, including LVH [33]. More importantly, many studies suggested that obesity and hypertension might have additive effects in increasing the risk of cardiovascular diseases during long-term follow-up, and the real risk in obese hypertensive patients was the long-term effects of obesity-related metabolic diseases on cardiovascular risk, such as obesity-related dyslipidemia. Therefore, decrease of body weight and blood pressure could be effective strategies for inhibiting LVH. In the present study, we found that HQQR treatment for 10 weeks could significantly inhibit LVH and decrease body weight, Lee index and BP**.** Consistent with the above results, HQQR treatment for 10 weeks could improve glucolipid metabolism evidenced by decreasing the level of TC, TG, LDL-C, FBG, HOMA-IR index and increasing the level of HDL-C in OBH rats, which could be helpful to suppress LVH of OBH rats.

Sirtuin 1 (SIRT1), a survival factor related to lifespan prolongation, has been proved to deacetylate the lysine residues [34]. Mitochondrial biogenesis is a necessary physiological activity to maintain mitochondrial function and an important process to regulate the expression of mitochondrial genes and proteins. Activation of PGC-1α deacetylation can drive all aspects of mitochondrial biogenesis, including activating respiratory chain and enhancing the respiratory ability of mitochondria. Reduction of the mtDNA copy number is considered to be an indicator for impaired mitochondrial biogenesis [35]. Previous studies have confirmed that the disorder of mitochondrial biogenesis was closely interconnected with cardiac hypertrophy and hypertension [36]. In OBH rats, hypertrophy stimulator such as angiotensin II (Ang II) can cause damage of the mitochondrial electron transport chain and subsequently induce the excessive production of ROS in mitochondria. Increase of oxidative stress level induces the disturbance of mitochondrial biogenesis, leading to cardiomyocyte injury and hypertrophy. Sai et al*.* found that resveratrol could alleviate cardiac dysfunction in diabetic cardiomyopathy mice by the activation of SIRT1/PGC-1α deacetylation [37]. In our study, we found that HQQR treatment for 10 weeks in OBH rats could up-regulate the expression of COX1 and ATPase6, increase Mn-SOD activity and reduce the damage of mitochondrial oxidative stress via SIRT1/PGC-1α deacetylation. Additionally, HQQR administration for 10 weeks in OBH rats could increase energy supply evidenced by the increased myocardial ATP content. Furthermore, HQQR could increase mitochondrial biogenesis evidenced by increasing mtDNA copy number and up-regulating the expression of NRF1 and TFAM, which was also closely related to SIRT1-mediated deacetylation of PGC- 1α. These data strongly indicated that HQQR had beneficial effects on myocardial mitochondrial function in OBH rats.

In conclusion, we demonstrated that HQQR treatment for 10 weeks could lose weight, decrease Lee index, lower BP and improve glucolipid metabolism in OBH rats, which could be associated with the effects on suppressing LVH. Importantly, HQQR could improve mitochondrial function through SIRT1/PGC-1α deacetylation pathway, including increasing myocardial energy supply, improving the ability of antioxidant stress and maintaining normal mitochondrial biogenesis to alleviate LVH in OBH rats.

## Abbreviations

Ang II: angiotensin II
DBP: diastolic blood pressure
ETC: electron transport chain
FBG: fasting blood glucose
FIN: fasting insulin
HDL-C: high-density lipoprotein-cholesterol
HQQR: HuoXue QianYang QuTan Recipe
LDL-C: low-density lipoprotein-cholesterol
LVH: left ventricular hypertrophy
OBH: obesity hypertension
SBP: systolic blood pressure
SIRT1: Sirtuin 1
TC: total cholesterol
TG: total triglycerides
